# Production of the Marine Carotenoid Astaxanthin by Metabolically Engineered *Corynebacterium glutamicum*

**DOI:** 10.3390/md14070124

**Published:** 2016-06-30

**Authors:** Nadja A. Henke, Sabine A. E. Heider, Petra Peters-Wendisch, Volker F. Wendisch

**Affiliations:** Genetics of Prokaryotes, Faculty of Biology & CeBiTec, Bielefeld University, Bielefeld D-33615, Germany; n.henke@uni-bielefeld.de (N.A.H.); saeheider@aol.com (S.A.E.H.); petra.peters-wendisch@uni-bielefeld.de (P.P.-W.)

**Keywords:** astaxanthin production, carotenoids, genome-reduced *Corynebacterium glutamicum*, systematic approach, metabolic engineering

## Abstract

Astaxanthin, a red C40 carotenoid, is one of the most abundant marine carotenoids. It is currently used as a food and feed additive in a hundred-ton scale and is furthermore an attractive component for pharmaceutical and cosmetic applications with antioxidant activities. *Corynebacterium glutamicum*, which naturally synthesizes the yellow C50 carotenoid decaprenoxanthin, is an industrially relevant microorganism used in the million-ton amino acid production. In this work, engineering of a genome-reduced *C. glutamicum* with optimized precursor supply for astaxanthin production is described. This involved expression of heterologous genes encoding for lycopene cyclase CrtY, β-carotene ketolase CrtW, and hydroxylase CrtZ. For balanced expression of *crtW* and *crtZ* their translation initiation rates were varied in a systematic approach using different ribosome binding sites, spacing, and translational start codons. Furthermore, β-carotene ketolases and hydroxylases from different marine bacteria were tested with regard to efficient astaxanthin production in *C. glutamicum*. In shaking flasks, the *C. glutamicum* strains developed here overproduced astaxanthin with volumetric productivities up to 0.4 mg·L^−1^·h^−1^ which are competitive with current algae-based production. Since *C. glutamicum* can grow to high cell densities of up to 100 g cell dry weight (CDW)·L^−1^, the recombinant strains developed here are a starting point for astaxanthin production by *C. glutamicum*.

## 1. Introduction

Carotenoids are natural pigments with yellow-to-red coloring properties, found ubiquitously in plants, algae, fungi, and bacteria. These pigments form a subfamily of the large and diverse group of terpenoids with more than 55,000 different structures. Terpenoids are natural secondary metabolites composed of isoprene units, which typically exhibit flavoring, fragrance and coloring properties. Carotenoids and their derivatives have become more and more important for the health care industry due to their beneficial effects on human and animal health and their possible pharmaceutical, medical, and nutraceutical applications. For example, carotenoids are suggested to have beneficial effects on the human immune system and to protect against degenerative diseases and cancer [1,2,3]. Astaxanthin is a marine, red, cyclic C40 carotenoid and the third most important carotenoid on the global market after β-carotene and lutein, with a predicted sales volume of 670 metric tons valued at 1.1 billion US$ in 2020 [4]. Currently, astaxanthin is primarily used as a food and beverage colorant, in animal feed and in nutraceuticals [5] with e.g., an annual demand of 130 tons for coloration of poultry, salmon, lobster and fish [6]. Astaxanthin shows the strongest hitherto demonstrated anti-oxidant effect due to its keto and hydroxy groups at 4,4'- and 3,3'-beta-ionone ring positions, respectively. Those functional groups result in a more polar nature of astaxanthin and explain its unique antioxidative properties [7]. Furthermore, astaxanthin can be esterified which leads to increased stability [8]. Therefore, the demand for astaxanthin is particularly rising in the health sector [5]. Astaxanthin has been described to promote skin health and to have potential anti-aging effect [9]. Moreover, it alleviates the fatigue, inflammation, and aging of the eye [10,11,12]. Astaxanthin has a positive effect on blood rheology and potential antihypertensive properties, which makes it interesting for therapy of cardiovascular diseases [13,14]. Its wide potential for the reduction of inflammation also promotes the immune system functions [15]. In addition, astaxanthin was reported to have a positive impact on muscle recovery when used as a nutritional supplement [16].

Although the chemical synthesis of astaxanthin from petrochemical precursors is so far more cost-efficient and therefore dominates the market [17], consumer demand for naturally produced carotenoids is increasing [18]. Synthetic astaxanthin is often a mixture of *R*- and *S*-enantiomers and, thus, inferior to natural-based astaxanthin [19] and not suitable as a neutraceutical supplement without further complex and cost-intensive purification steps before application. Consequently, the demand for an efficient, environmentally friendly production of natural astaxanthin, and carotenoids in general, by microbial hosts is on the rise [20,21,22].

*C. glutamicum* is a Gram-positive soil bacterium with a long biotechnological history: its relevance goes back to the 1950s when it was first discovered as a natural glutamate producer [23]. Over centuries it has been used for the million-ton scale production of different amino acids for the feed and food industry. Moreover, its potential for biotechnological application has been further exploited [24]: besides amino acids, e.g., diamines [25], alcohols [26], and terpenoids [27,28] can be produced by engineered *C. glutamicum*. This bacterium has the ability to grow aerobically on a variety of carbon sources like glucose, fructose, sucrose, mannitol, propionate, and acetate [29,30]. In addition, it has been engineered to grow with alternative carbon sources such as glycerol [31], pentoses [32], amino sugars [33,34], β-glucans [35], and starch [36]. *C*. *glutamicum* is pigmented due to synthesis of the C50 carotenoid decaprenoxanthin and its glucosides. Its potential to produce carotenoids has been explored over recent years [28,37,38,39]. The carotenogenic pathway of *C*. *glutamicum* was identified [40] and several metabolic engineering strategies were applied to convert this biotechnologically established bacterium into a carotenoid producer [41,42].

In order to enable C40 carotenoid production by *C. glutamicum*, the conversion of lycopene to decaprenoxanthin needs to be prevented by deletion of the genes encoding lycopene elongase and ε-cyclase. As consequence of deletion of the lycopene elongase encoding gene *crtEb*, the cells exhibited a slight red color due to accumulation of the intermediate lycopene [37]. Additional overexpression of the endogenous genes *crtE*, *crtB*, and *crtI* in *C. glutamicum* Δ*crtEb* intensified the red phenotype as conversion of GGPP to the red chromophore lycopene was improved. Thereby, the lycopene content could be increased 80 fold with 2.4 ± 0.3 mg·(g·CDW)^−1^ and showed for the first time enhanced C40 carotenoid production in *C. glutamicum* [37]. Heterologous expression of *crtY* from *Pantoea ananatis* (*crtY_Pa_*) in a lycopene accumulating platform strain led to the production of the orange pigment β-carotene. Zeaxanthin was accumulated when *crtZ* from *P. ananatis* (*crtZ_Pa_*) was expressed in addition [38]. Furthermore, carotenoid biosynthesis was improved by enhancing the precursor supply, which was accomplished by overexpression of the *dxs* gene encoding the enzyme for the initial condensation of pyruvate and GAP in the MEP-pathway [42].

**Figure 1:**
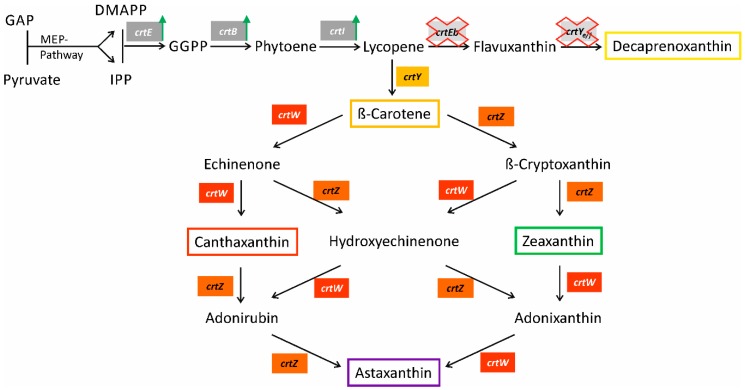
Scheme of C40 cyclic carotenoid biosynthesis in recombinant *C. glutamicum*. The biosynthesis of C40 cyclic carotenoids derived from precursor molecules dimethylallyl pyrophosphate (DMAPP) and isopentenyl pyrophosphate (IPP) is illustrated. Genes are shown next to the reaction catalyzed by the encoded enzyme (*crtE*: Prenyl transferase, *crtB*: Phytoene synthase, *crtI*: Phytoene desaturase, *crtEb*: Lycopene elongase, *crtYe/f*: C45/50 carotenoid ε-cyclase, *crtY*: Lycopene β-cyclase, *crtZ*: β-Carotene hydroxylase (3,3'-beta-ionone ring hydroxylase), *crtW*: β-Carotene ketolase (4,4'-beta-ionone ring ketolase). Endogenous genes are shown in grey boxes and their overexpression indicated by green arrows. Heterologous genes are highlighted in colored boxes.

In this study production of the marine carotenoid astaxanthin by *C. glutamicum* was developed using a β-carotene producing strain (Figure 1). Two strategies were followed: (i) the implementation of a combinatorial gene assembly for *crtW_Ba_* and *crtZ_Pa_* to optimize the ratio of enzyme quantities (ketolase and hydroxylase) by variation of translation initiation rates (TIR) based on different ribosome binding sites, spacing lengths, and translation start codons and (ii) the use of alternative *crtW* and *crtZ* genes from marine and non-marine prokaryotes in a two-vector system in order to find enzymes with higher activities or affinities for the intermediates of the astaxanthin biosynthesis pathway (Figure 1). Combined expression of *crtW* and *crtZ* from the marine bacterium *Fulvimarina pelagi* yielded a *C. glutamicum* strain producing astaxanthin as the major carotenoid in shaking flasks with productivities of up to 0.35 mg·L^−1^·h^−1^.

## 2. Results

### 2.1. Construction of a β-Carotene Producing C. glutamicum Base Strain

*C. glutamicum* was metabolically engineered for plasmid-independent lycopene overproduction (Table 1). Chromosomal integration of the synthetic operon *crtEBI* under the control of the endogenous promoter of the gene coding for the translational elongation factor (*P_tuf_*) in the *crtY_e_Y_f_Eb* deletion mutant of *C. glutamicum* MB001 (LYC3) [37] was performed in order to improve the expression of prenyltransferase CrtE, phytoene synthase CrtB and phytoene desaturase CrtI encoding genes. Thereby, the flux from the precursor molecules IPP and DMAPP to lycopene was enhanced and an 8-fold higher lycopene titer resulted for strain LYC4. When *dxs*, encoding the first enzyme of the MEP-pathway, was additionally overexpressed by chromosomal exchange of its natural promoter by the strong promoter *P_tuf_*, the lycopene titer was further improved by 34% and the respective strain LYC5 produced 0.43 ± 0.02 mg·(g·CDW)^−1^ (Table 1).

Strain LYC5 was converted to a β-carotene producing strain (Table 2) by heterologous expression of the lycopene β-cyclase gene *crtY* from *P. ananatis.* Plasmid-borne expression of *crtY* under the control of the isopropyl β-D-1-thiogalactopyranoside (IPTG) inducible *tac* promoter (pEKEx3_*crtY_Pa_*) allowed for β-carotene production. Constitutive expression of *crtY* under control of the *P_tuf_* promoter from the newly constructed expression and shuttle vector pSH1 resulted in a comparable production titer. Similarly, a β-carotene titer of 6.5 mg·g^−1^ was achieved by BETA3, a strain having *crtY_Pa_* under the control of *P_tuf_* integrated into the genome of *C. glutamicum* strain LYC5 (Table 2).

### 2.2. Design of the Combinatorial Gene Assembly and Library Construction for Engineering Astaxanthin Production in C. glutamicum

**Figure 2:**
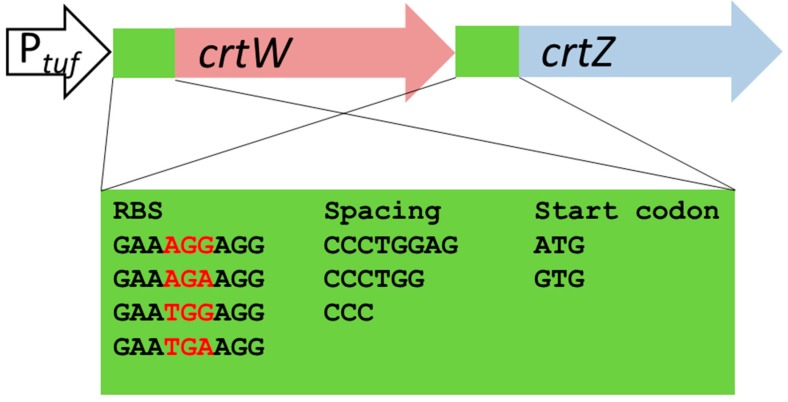
Combinatorial gene assembly for varied translation initiation of β-carotene ketolase and hydroxylase genes. Combinations of different RBS sequences (differences given in red letters), translation start codons (ATG/GTG) and spacers (3, 6 or 8 bp in length) between them are highlighted in a green box.

Metabolic flux in a synthetic pathway may require well-adjusted activities of the enzymes involved. Prediction of the flux from gene expression is rather difficult, hence, a combinatorial gene assembly was used to screen for balanced expression of the β-carotene ketolase and β-carotene hydroxylase encoding genes with respect to astaxanthin production. Since *crtY* from *P. ananatis* has previously been expressed successfully in *C. glutamicum* for production of β-carotene, the β-carotene hydroxylase *crtZ* gene from this organism was chosen. However, *P. ananatis* lacks β-carotene ketolase, and therefore the β-carotene ketolase gene *crtW* from *Brevundimonas aurantiaca* was used, which on the contrary lacks a *crtZ* gene. *CrtW* from *B. aurantiaca* and *crtZ* from *P. ananatis* were combined in an artificial operon under the control of the constitutive *P_tuf_* promoter in the vector pSH1. Gene expression was varied by combining different ribosome-binding sites (RBS) and start codons separated by spacers of different lengths (Figure 2). The theoretical translation initiation rates were calculated using the RBS calculator [43] and ranged from 14 to 33,626 for *crtW* and from 40 to 30,731 for *crtZ*. A library of combinatorially assembled *crtW* and *crtY* genes was generated and the constructed library of pSH1_*crtW_Ba__crtZ_Pa_* plasmids was used to transform the β-carotene accumulating strain *C. glutamicum* BETA1.

For each gene four different RBS, three different spacer lengths, and two different translational start codons were chosen. These were introduced by the forward primers and equimolar mixture of these primers and one reverse primer by PCR. The resulting DNA products were gel-extracted and combined by cloning via Gibson Assembly [44] in pSH1. Thus, theoretically 24 different constructs per gene resulted (Figure 2). With this approach 576 different combinations of *crtW* and *crtZ* genes are theoretically possible and the event of creating a specific combination of the two genes follows the Poisson distribution [45] with a probability of 1/576 (Equation (1)). To cover with 99% probability that a single specific combination is present at least once in the library, approximately 2650 clones are required (Equation (1)). The necessary number of transformants for creating a library with each of the 576 combinations can be calculated employing the path rules [45]. For creating a library that includes each of the 576 specific combinations at least once with a 99% probability, approximately 6315 transformants are required (Equation (2)). Preliminary experiments showed that correct assembling of an insert with the restricted vector via Gibson assembly occurs in about 90% of the events. Consequently, the number of transformants had to be corrected by multiplication by 1.11, thus, a minimum of 7000 transformants had to be screened.

(1) $$p_{\lambda }\left(k\right)=\frac{\lambda^{k}}{k!}*e^{-\lambda }$$

Equation (1): Poisson distribution. λ = *n* * *p*; *n*: library size; *p*: probability of one specific gene assembly of *crtW* and *crtZ*, *k*: number of one specific gene assembly in library with size *n*.

(2) $$p_{all}\left(k\geq 1\right)={\left(1-e^{-\lambda }\right)}^{N}$$

Equation (2): Path rules. λ = *n* * *p*; *n*: library size; *p*: probability of one specific gene assembly of *crtW* and *crtZ*, *k*: number of one specific gene assembly in library with size *n*; *N*: number of possible gene assemblies.

Around 8000 transformants were visually color-screened on plates and 46 colonies with different colors ranging from yellow to red were selected for further analysis. The plasmid DNA was isolated and sequenced to identify the sequences (RBS, spacer, translational start codon) of *crtW* and *crtZ.* The set of 46 transformants represented 20 of the 24 possible variants for *crtW* and 19 of 24 variants of *crtZ*. Furthermore, three plasmids harbored only the *crtW* gene and two plasmids harbored only the *crtZ* gene.

### 2.3. Combinatorial Engineering Covered Vastly Different Astaxanthin, β-Carotene, Zeaxanthin and Canthaxanthin Titers

**Figure 3:**
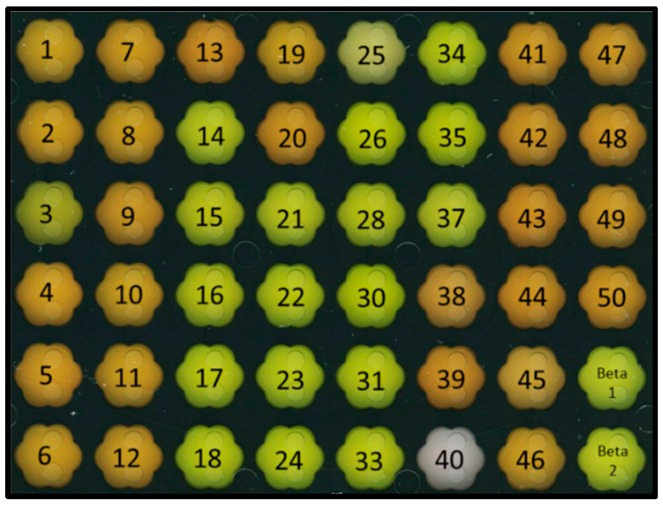
COMB strains expressing *crtW* from *B. aurantiaca* and *crtZ* from *P. ananatis* with varied translation initiation signals after growth in the Biolector micro fermentation system. Color phenotypes of 46 different COMB strains and the parental strain BETA1 (bottom right) after 24 h of cultivation.

To evaluate which of the gene combinations was best in terms of astaxanthin production, the 46 selected transformants referred to as COMB strains, were characterized with respect to carotenoid production. After growth in CGXII minimal medium with 100 mM glucose, appropriate antibiotics and 1 mM IPTG in a Biolector micro fermentation system (Figure 3), carotenoids were quantified by HPLC using standards for β-carotene, canthaxanthin, zeaxanthin, and astaxanthin.

**Figure 4:**
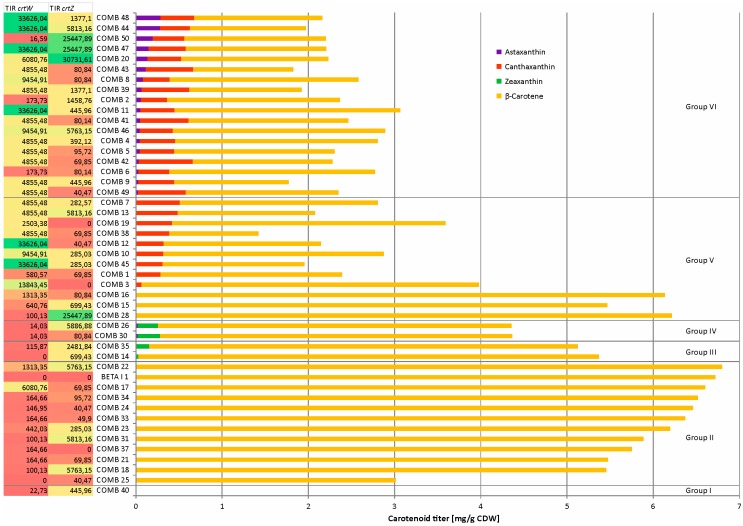
Carotenoid profiles and calculated translational initiation rates (TIRs) for *C. glutamicum* strains expressing *crtW* from *B. aurantiaca* and *crtZ* from *P. ananatis* with varied translation initiation signal. TIRs were calculated by applying the RBS calculator tool [46] on the mRNA sequence. TIRs were classified as follows: TIRs <200: low; 200 < TIRs < 2000: medium; TIRs >2000: high. Production of β-carotene, zeaxanthin, canthaxanthin and astaxanthin was determined after 24 h of cultivation in CGXII + 100 mM glucose in Biolector micro fermenter.

As expected, the parental strain BETA1 (Figure 3) produced β-carotene (6.7 mg·(g·CDW)^−1^), but no further carotenoids. The 46 COMB strains could be categorized in six groups according to their carotenoid production profiles (group I: only lycopene, group II: only β-carotene, group III: β-carotene and zeaxanthin, group IV: β-carotene, zeaxanthin and astaxanthin, groupV: β-carotene and canthaxanthin, group VI: β-carotene, canthaxanthin and astaxanthin; Figure 4). For all COMB strains, the TIRs were calculated with the RBS calculator tool [41], which takes (amongst others) the free binding energy of the RBS and the 16S rRNA into consideration as well as the free energy of secondary structures of the mRNA itself.

COMB 40 (Figure 4) produced none of the cyclic carotenoids, but about as much lycopene (0.39 mg·(g·CDW)^−1^) as LYC5 (0.5 ± 0.1 mg·(g·CDW)^−1^), the parental strain of BETA1. Sequencing of pEKEx3_*crtY_Pa_* isolated from COMB 40 revealed a deletion of 11 base pairs in the coding region of *crtY*, hence, β-carotene production was not possible in this strain. By contrast, the other 45 strains produced β-carotene with a titer of at least 1 mg·(g·CDW)^−1^ (Figure 4). For about 24% of the strains, β-carotene was the only cyclic carotenoid being produced. In these cases, the calculated TIRs of *crtW* and/or *crtZ* were rather low (less than 200 for at least one gene; Figure 4). Zeaxanthin, one of the intermediates in the pathway towards astaxanthin, was detected in only four strains (COMB 14, COMB 26, COMB 30, and COMB 35) and these strains exhibited very diverse TIRs for *crtZ* (from 81 to 5887) (Figure 4). The highest production of zeaxanthin was detected in group IV for strain COMB 30 with 0.3 mg·(g·CDW)^−1^, although this strain possessed a low TIR for *crtZ*. The highest titers of canthaxanthin and astaxanthin were observed among the strains of the large group VI (39%) and these strains co-produced β-carotene along with canthaxanthin and astaxanthin (Figure 4). The intermediate canthaxanthin was detected in 30 strains with strain COMB 42 showing the highest titer for canthaxanthin (0.6 mg·(g·CDW)^−1^; Figure 4). In average, the TIR for *crtW* of these strains was high (10,299). Astaxanthin was identified in 20 of the 46 strains, but only two strains, COMB 44 and COMB 48, exhibited reasonably high astaxanthin yields (approximately 0.3 mg·(g·CDW)^−1^). These two strains exhibited high *crtW* TIRs (33,626) and medium to high *crtZ* TIRs (5813 and 1377) (Figure 4).

In general, it was found that the higher the TIR of *crtW* the higher was the astaxanthin production, with three exceptions, COMB 11, COMB 12, and COMB 45 (Figure 4). In the latter three strains, however, the TIRs for *crtZ* were 3 to 145-fold lower than in the best astaxanthin producing strains COMB 44 and COMB 48. For strains COMB 44 and COMB 48, a spacing length of six base pairs, the RBS sequence GAAAGGAGG, and the translation start codon ATG was found for *crtW*. The *crtZ* gene in COMB 44 showed the consensus RBS sequence, a spacer length of eight base pairs and ATG as translational start codon. The *crtZ* gene variant of COMB 48 had a slightly lower TIR and possessed the RBS sequence GAAAGAAGG, six base pairs of spacing and ATG as start codon.

Three strains (COMB 37, COMB 3 and COMB 19) did not express *crtZ* due to an incorrect gene assembly. Strains COMB 3 and COMB 19 accumulated canthaxanthin besides β-carotene, while COMB 37, which also showed a low TIR for *crtW*, only accumulated β-carotene (Figure 4). Strains COMB 25 and COMB 14 did not express *crtW* due to an incorrect gene assembly. While COMB 25 only produced β-carotene probably because of a very low TIR for *crtZ* (Figure 4), strain COMB 14 produced zeaxanthin besides β-carotene.

Taken together, widely varied carotenoid production was represented by the library, but none of the combinations tested yielded high astaxanthin product levels.

### 2.4. In Silico Analysis of the Carotenogenic Genes crtZ and crtW from Marine and Non-Marine Bacteria for Heterologous Expression in C. glutamicum

In the above described experiments the bacteria *B. aurantiaca* and *P. ananatis* were chosen as sources for *crtW* and *crtZ*, respectively, although not producing astaxanthin themselves. *B. aurantiaca* lacks *crtZ*, but possesses the *crtG* gene coding for a 2,2'-beta-ionone ring hydroxylase and produces canthaxanthin and 2-hydroxycanthaxanthin as main carotenoids. *P. ananatis* lacks *crtW* and produces glycosylated zeaxanthin involving CrtZ. Thus, on the basis of available genome sequences, reported carotenoid production and biological diversity, four alternative prokaryotic natural carotenoid producers were selected as donors for *crtW* and *crtZ*. Since *crt* genes of *Brevundimonas* species were reported to show a high potential for heterologous carotenoid productions [47] two different *Brevundimonas* strains were selected: *Brevundimonas vesicularis*, a non-marine bacterium suggested to be a suitable gene donor for astaxanthin production [47,48], and as alternative *Brevundimonas bacteroides* [49]. The marine bacterium *Fulvimarina pelagi* was chosen due to its promising brownish-yellow color as a consequence of carotenoid accumulation [50] and the evolutionary distance to *Brevundimonas*. In addition, the red-pigmented marine bacterium *Sphingomonas astaxanthinifaciens* was selected since experimental evidence that astaxanthin is the major carotenoid produced by this bacterium has been reported [51,52].

The organization of carotenogenic gene clusters of the considered donors *B. aurantiaca*, *B. bacteroides*, *B. vesicularis*, *F. pelagi*, *P. ananatis*, and *S. astaxanthinifaciens* was analyzed on the basis of the partly available genome sequences/contigs or scaffolds in GenBank: the carotenoid gene cluster of *B. bacteroides*, an orange-red pigmented bacterium, comprises *crtW* and *crtZ* as well as the gene *idi* encoding the IPP isomerase of the MEP-pathway and several other genes encoding for enzymes of the astaxanthin biosynthesis pathway, however, a *crtE* gene is not present in its genome. The genome of *B. vesicularis* DC263, a red-pigmented soil bacterium, possesses a large carotenoid gene cluster with 10 coding sequences, eight of which encode enzymes for the biosynthesis pathway of astaxanthin or the terpenoid precursors IPP and DMAPP. In addition, a second hydroxylase CrtG is encoded, which is responsible for further hydroxylation of astaxanthin leading to 2-hydroxyastaxanthin. Carotenogenic genes of *F. pelagi*, a Mn(II)-oxidizing bacterium [53], are found in at least four different loci of the genome. Genes encoding for enzymes of the astaxanthin biosynthesis and glycosylation as well as enzymes for the spirilloxanthin biosynthesis (CrtC, CrtD, CrtF) are present. Furthermore, two genes coding for an ABC-transporter and a MFS-transporter are located next to the carotenogenic genes *crtZ* and *crtY*. *S. astaxanthinifaciens*, producing astaxanthin and its glycosides, has at least two carotenoid gene clusters in its genome also including farnesyl pyrophosphate synthase. Moreover, a gene encoding a putative carotenoid transporter is located in this cluster.

### 2.5. High Astaxanthin Production by C. glutamicum Strains Expressing crtW and crtZ from F. pelagi

β-Carotene ketolase and hydroxylase genes (*crtW* and *crtZ*, respectively) from *B. aurantiaca*, *B. bacteroides*, *B. vesicularis*, *F. pelagi*, *P. ananatis*, and *S. astaxanthinifaciens* were expressed in the plasmid-free β-carotene overproducing *C. glutamicum* strain BETA4. The affinities of the β-carotene ketolases and hydroxylases for the various substrates and intermediates of the branched astaxanthin biosynthesis pathway may vary and it is conceivable that astaxanthin production proceeds e.g., only via canthaxanthin or only via zeaxanthin. However, also various other routes via hydroxyechinenone are possible (Figure 1). Thus, in a first step only either *crtW* or *crtZ* was expressed in the parental strain BETA4 that produced ~12 mg·(g·CDW)^−1^ β-carotene with a productivity of ~3.4 mg·L^−1^·h^−1^ in 24 h of cultivation and a growth rate of 0.32 ± 0.01 h^−1^. Zeaxanthin accumulated (0.52 and 1.1 mg·(g·CDW)^−1^, respectively) when *crtZ* from *P. ananatis* or *F. pelagi* were expressed (data not shown). Canthaxanthin accumulated (0.34 to 1.0 mg·(g·CDW)^−1^) when *crtW* from *S. astaxanthinifaciens*, *F. pelagi* or *B. aurantiaca* were expressed, while only traces were observed as consequence of expression of *crtW* from *B. bacteroides* or *B. vesicularis* (data not shown). Thus, *crtW* from *S. astaxanthinifaciens*, *F. pelagi* or *B. aurantiaca* and *crtZ* from *P. ananatis* or *F. pelagi* appeared suitable for further analysis.

Subsequently, combinations of *crtW* from *S. astaxanthinifaciens* and *B. aurantiaca* with *crtZ* from *F. pelagi* were co-expressed in strain BETA4 using the two expression vectors pSH1 and pEC-XT99A. In addition, the *crtW* and *crtZ* genes from species known to synthesize astaxanthin (*B. bacteroides*, *B. vesicularis*, *F. pelagi*, and *S. astaxanthinifaciens*) were co-expressed in BETA4. Carotenoids of these strains were extracted and analyzed in the stationary growth phase 24 h after inoculation. Transformants harboring the genes from *B. vesicularis* grew poorly and were not analyzed further.

Co-expression of *crtW* and *crtZ* from *B. bacteroides* and *crtW* and *crtZ* from *S. astaxanthinifaciens* genes led to less than 0.1 mg·(g·CDW)^−1^ of astaxanthin (Table 3). Strains expressing *crtZ* from *F. pelagi* in combination with *crtW* from *S. astaxanthinifaciens, B. aurantiaca,* and *F. pelagi* produced about 0.7, 1.7 and 1.6 mg·(g·CDW)^−1^ of astaxanthin, respectively (Table 3). In all strains except for BETA4(pSH1_*crtW_Fp_*)(pEC-XT_*crtZ_Fp_*), β-carotene was the major carotenoid (~2 mg·(g·CDW)^−1^). *C. glutamicum* BETA4 co-expressing *crtW* and *crtZ* from *F. pelagi* (subsequently named ASTA1) produced astaxanthin as main carotenoid (1.6 ± 0.3 mg·(g·CDW)^−1^) and accumulated little canthaxanthin and β-carotene (0.1 ± 0.1 and 0.3 ± 0.1 mg·(g·CDW)^−1^, respectively) as side products (Table 3). This strain produced astaxanthin with a volumetric productivity of 0.4 ± 0.1 mg·L^−1^·h^−1^ in shaking flasks with a growth rate of 0.29 ± 0.05 h^−1^.

## 3. Discussion

In this study, *Corynebacterium glutamicum* was engineered for the production of the marine carotenoid astaxanthin. *C. glutamicum* grows fast to high cell densities [54] and, thus, is suitable for production of carotenoids and other compounds that are stored within the cell. Here, *C. glutamicum* was shown to produce β-carotene to about 12 mg·(g·CDW)^−1^ within 24 h at a volumetric productivity of about 3.4 mg·L^−1^·h^−1^. Growth and production of carotenoids by *C. glutamicum* is monophasic and strains BETA4 and ASTA1 showed growth rates of 0.32 ± 0.01 h^−1^ and 0.29 ± 0.05 h^−1^, respectively. This is in contrast to biphasic growth/production of carotenoids e.g., by the alga *Haematococcus pluvialis* [55]. As a consequence, the volumetric productivity for β-carotene exceeds that reported for the industrially used microalga *Dunaliella bardawil* [56] or the yeast *Saccharomyces cerevisiae* [57] by about a factor of three.

Combined expression of the genes coding for β-carotene ketolase and hydroxylase from microorganisms that do not synthesize astaxanthin (*B. aurantiaca* and *P. ananatis*) in a β-carotene producing *C. glutamicum* led to astaxanthin production. However, astaxanthin was not the main carotenoid being produced. Since a balanced expression of the β-carotene ketolase and hydroxylase genes are essential for an efficient astaxanthin production [48,58] we assumed that the activities of the respective enzymes in the tested recombinants were not matched. Therefore, translation initiation rates of the respective genes, *crtW* and *crtZ*, were varied in a combinatorial approach. However, a strict correlation between TIR and production titers was not observed. As tendencies, the lower the TIRs of both *crtW* and *crtZ* the lower were the canthaxanthin and astaxanthin titers, and the higher the TIR of *crtW* the higher were astaxanthin titers (Figure 4).

In *E. coli* astaxanthin biosynthesis from β-carotene was reported to proceed more efficiently via zeaxanthin rather than canthaxanthin since ketolated intermediates did not accumulate [48,58]. Both ketolase and hydroxylase compete for their substrates and accept β-carotene as well as canthaxanthin and zeaxanthin, respectively, as substrates [59,60]. Independently induced expression of *crtZ* from *P. ananatis* and *crtW148* of *Nostoc puntiforme* PC73102 revealed that hydroxylation occurred fast with β-carotene, echinenone, adonirubin, and canthaxanthin [58]. In their system, CrtW148 was identified as the limiting step in conversion of zeaxanthin to astaxanthin [58]. Expression of *crtZ* from *P. ananatis* in β-carotene producing *C. glutamicum* also yielded zeaxanthin [38] as did expression of *crtZ* from *F. pelagi* in this study (data not shown). Varying expression levels of *crtW_Ba_* and *crtZ_Pa_* led to accumulation of zeaxanthin only if TIR for *crtW_Ba_* was low (Figure 4). On the other hand, canthaxanthin accumulated as intermediate typically if TIR of *crtW_Ba_* was medium to high (Figure 4). Canthaxanthin accumulation may be explained best by the assumption that β-carotene ketolase CrtW from *B. aurantiaca* did not accept the non-natural substrate zeaxanthin well. It is likely that astaxanthin production by this approach was not only limited by an imperfect match between expression levels of the β-carotene ketolase and hydroxylase genes, but rather by imperfect compatibility of the substrate spectra of the chosen β-carotene ketolase and hydroxylase enzymes.

Consequently, *crtW* and *crtZ* genes from marine and non-marine bacteria known to synthesize astaxanthin were examined in the second approach. Astaxanthin was produced in combinations of CrtZ from the marine bacterium *F. pelagi* and CrtW from either *F. pelagi*, *S. astaxanthinifaciens* or *B. aurantiaca*. *F. pelagi* was isolated from ocean surface water, an aerated environment at least transiently exposed to high solar radiation [45]. It is hypothesized that carotenoids play an important role as antioxidants for survival of *F. pelagi* under these conditions [50]. Analysis of the codon usage of *crtW* and *crtZ* from *F. pelagi* revealed a good fit to the codon usage of *C. glutamicum*, which is in compliance with the achieved astaxanthin titers of the recombinants. Co-expression of *crtW* from *B. aurantiaca* and *crtZ* from *F. pelagi* led to comparable astaxanthin titers, but considerable β-carotene amounts accumulated as side-product (Table 3), co-expression of *crtW* and *crtZ* from *F. pelagi*, instead, yielded astaxanthin as major carotenoid (80%; Table 3).

As compared to β-carotene production of about 12 mg·(g·CDW)^−1^ by the parent strain BETA4, the astaxanthin titers were at least seven fold lower (Table 3). Thus, conversion of β-carotene to astaxanthin is incomplete; however, other carotenoids besides canthaxanthin and residual β-carotene did not accumulate to significant titers (data not shown and Table 3). The partial conversion of β-carotene to astaxanthin may, thus, indicate that astaxanthin and/or intermediate(s) of its biosynthesis are inhibitory. This is in line with our finding that overexpression of only *crtW* from *F. pelagi* resulted in 0.5 mg·(g·CDW)^−1^ canthaxanthin and 1.7 mg·(g·CDW)^−1^ remaining β-carotene. Similarly, overexpression of only *crtZ* yielded 1.1 mg·(g·CDW)^−1^ zeaxanthin and 5.6 mg·(g·CDW)^−1^ β-carotene remained. Similarly, heterologous expression of *crtW148* and *crtZ* in the β-carotene-producing *E. coli* strain reduced the overall formation of carotenoids, indicating that the formation of the carotenoid precursors were affected [58].

High product purities and titers are beneficial for downstream processing. The astaxanthin producing *C. glutamicum* strain overexpressing *crtW* and *crtZ* from *F. pelagi* accumulated astaxanthin (about 1.6 mg·(g·CDW)^−1^) as major (about 80%) carotenoid. The fact that little β-carotene and canthaxanthin accumulated (about 0.3 and 0.1 mg·(g·CDW)^−1^, respectively) may be an important advantage for downstream processing. Nevertheless, higher product purities can be obtained by algae with 95% of total carotenoids being astaxanthin [58]. Purification of astaxanthin from the cell walls of algae and red yeasts is challenging since algae like *H. pluvialis* accumulate astaxanthin in response to stress and heavily walled cysts are formed in the red stage [55]. Extraction of carotenoids from microalgae does not only require the removal of chlorophyll [61], but also efficient cell breakage technology [55]. Ethoxyquin or other antioxidants are added to the cells in order to minimize oxidation of the carotenoids during drying and cracking [58]. Because of laborious and time-consuming extraction processes of astaxanthin from algal systems, its production by a prokaryotic host, *Escherichia coli*, has emerged for substitution [62]. It has to be noted that *H. pluvialis* produces esterified astaxanthin, which is more stable than the free form astaxanthin as it does not cross react with proteins and e.g., lipoproteins [8], and which is incorporated easier by marine animals [63]. But hydrolysis of the ester narrows the bioavailability of astaxanthin e.g., to salmon [64]. The rigid cell walls of the red yeast *X. dendrorhous* also requires cell breakage prior to astaxanthin extraction [65,66]. In contrast to that, a simple methanol-acetone extraction was sufficient to recover astaxanthin from *C. glutamicum* cells at lab scale.

The volumetric productivities of up to about 0.4 mg·L^−1^·h^−1^ obtained in simple shaking flask cultures by the recombinant *C. glutamicum* strains compare favorably with those reported for the commercially used production hosts such as the green microalgae *H. pluvialis* [55,67] and the red yeast *Xanthophyllomyces dendrorhous* (formerly *Pfaffia rhodozyma*) [6,68] under similar conditions as well as recombinant *E. coli* [58]. Under optimal conditions, astaxanthin titers obtained e.g., with *H. pluvialis* are very high (up to about 40 mg·(g·CDW)^−1^), but slow growth, biphasic growth (green stage) and production (red stage) properties and the low final biomass concentrations reduce the maximal volumetric productivity [55]. After the non-productive green phase (about 4 days), the volumetric productivity for astaxanthin in the red stage is about 1 mg·L^−1^·h^−1^ and can be maintained for extended periods [55]. Although astaxanthin product titers from red yeasts such as *X. dendrorhous* are generally lower than from algae [69], higher growth rates and easier cultivation conditions argue in favor of these yeasts [70]. After optimization of a glucose-based fed-batch process a volumetric productivity of about 5 mg·L^−1^·h^−1^ was achieved [65,71]. Can it be envisioned that comparably high volumetric productivities can be obtained using the recombinant *C. glutamicum* strains described here? In pressurized high-cell-density fed-batch cultivations *C. glutamicum* grows to biomass concentrations of about 220 g·CDW·L^−1^ within 24 h [54]. If this growth could be achieved with the *C. glutamicum* strains accumulating astaxanthin to titers of about 1.6 mg·(g·CDW)^−1^, theoretically volumetric productivities of about 14 mg·L^−1^·h^−1^ may be achieved. Future work focused on process intensification, however, needs to be performed in order to evaluate if scale-up to such high astaxanthin volumetric productivities can be realized with *C. glutamicum*.

## 4. Materials and Methods

### 4.1. Bacterial Strains, Media and Growth Conditions

The strains and plasmids used in this work are listed in Table 4. *C. glutamicum* ATCC 13032 was used as wild type (WT), for metabolic engineering the prophage-cured *C. glutamicum* MB001 [72] was used as platform strain. Pre-cultivation of *C. glutamicum* strains was performed in LB medium or LB with 50 mM glucose. For cultivation in CGXII medium [73], pre-cultivated cells were washed once with CGXII medium without carbon source and inoculated to an initial OD_600_ of 1. Glucose was added as carbon and energy source to a concentration of 100 mM. Standard cultivations of *C. glutamicum* were performed at 30 °C in a volume of 50 mL in 500 mL flasks with two baffles shaking at 120 rpm. The OD_600_ was measured in dilutions using a Shimadzu UV-1202 spectrophotometer (Duisburg, Germany). Alternatively, cultivations were performed in 1 mL volume in micro-titerplates at 1100 rpm at 30 °C using Biolector^®^ micro fermentation system (m2p-labs GmbH, Baesweiler, Germany). For cloning, *E. coli* DH5α was used as host and cultivated in LB medium at 37 °C. When appropriate, kanamycin, tetracycline or spectinomycin was added to concentrations of 25, 5, and 100 µg·mL^−1^, respectively. Gene expression was induced by addition of 1 mM IPTG, at inoculation of the main culture.

### 4.2. Recombinant DNA Work

Plasmids were constructed in *E. coli* DH5α from PCR-generated fragments (All-in HiFi, highQu, Kraichtal, Germany) and isolated with the Plasmid GeneJET Miniprep kit (Thermo Fisher Scientific, Schwerte, Germany). Oligonucleotides used in this study were obtained from Metabion (Planegg/Steinkirchen, Germany) and are listed in Table 5. Standard reactions like restriction, and PCR were performed as described previously [83]. Besides cloning by restriction, Gibson assembly was applied for the construction of plasmids [44]. If applicable, PCR products were purified using the PCR clean-up and gel extraction kit (Macherey-Nagel, Düren, Germany). For transformation of *E. coli* DH5α, the RbCl method was used and *C. glutamicum* was transformed via electroporation [84] at 2.5 kV, 200 Ω, and 25 µF. All cloned DNA fragments were confirmed by sequencing.

### 4.3. Construction of Expression Vector pSH1

The plasmid pSH1 was constructed based on the expression vector pVWEx1 [81]. The backbone of pSH1 was amplified from pVWEx1 omitting the *lacIq* and *P_tac_* region using the oligonucleotides pV1-fw and pV6962-rv (Table 5) with All-in HiFi polymerase (highQu, Kraichtal, Germany). The promoter of the *C. glutamicum tuf* gene (cg0587) was amplified using the primers pV_*P_tuf_*-fw and pV_*P_tuf_*-rv (Table 5). Both fragments were assembled using the Gibson method [44]. Vector sequence was confirmed via sequencing to exclude mutations.

### 4.4. Deletion and Exchenge Mutagenesis in the Genome of C. glutamicum

For targeted deletion of *cg0725*, which encodes a transcriptional regulator and is part of the carotenogenesis gene cluster of *C. glutamicum*, the suicide vector pK19*mobsacB* was used [82]. Genomic regions flanking *cg0725* were amplified from genomic DNA of *C. glutamicum* WT using primer pairs *cg0725*-A/B and *cg0725*-C/D (Table 5), respectively. Subsequently the purified PCR products were linked by crossover PCR using the primer pair *cg0725*-A/D (Table 5). The resulting amplificate was cloned into pK19*mobsacB* resulting in the construction of deletion vector pK19*mobsacB*-*cg0725* (Table 4). Deletion of *cg0725* via two-step homologous recombination as well as the selection for the first and second recombination events were carried out as described previously [85]. Successful removal of *cg0725* was verified by PCR analysis of the constructed mutant using primer pair *cg0725*-E/F (Table 5).

The integration of the synthetic operon *crtEBI* and the lycopene cyclase gene of *Pantoea ananatis crtY*, respectively, was conducted by using the suicide vector pK19*mobsacB* [82]. Operon *crtEBI* consists of the carotenogenic genes *crtE* (cg0723), *crtB* (cg0721) and *crtI* (cg0720) and was amplified from the expression vector pVWEx1-*crtEBI* [38] using the oligonucleotides *crtEBI*-Int5 and *crtEBI*-Int6. The *P_tuf_* promoter region was amplified using the oligonucleotides *crtEBI*-Int3/4 or *crtY*-Int3/4, respectively. Genomic regions flanking the selected insertion region were amplified from genomic DNA of *C. glutamicum* MB001 using primer pairs *crtEBI*-Int1/2 and *crtEBI*-Int7/8 for integration in the cgp2 cured region in the case of the *crtEBI* operon, or *crtY*-Int1/2 and *crtY*-Int7/8 for integration of *crtY* in the CGP1 cured region (Table 5), respectively. *CrtY* was amplified from genomic DNA of *P. ananatis* using the primer pair *crtY*-Int5/6. The purified PCR products were directly combined together with the plasmid by Gibson assembly [44]. The final assembly of the insert with linearized pK19*mobsacB* led to the construction of the respective integration vectors pK19*mobsacB*-Int*crtEBI* and pK19*mobsacB*-Int*crtY* (Table 4). The following integration of the operon by two-step homologous recombination was performed according to the deletion of genes. The integration in the cgp1 or cgp2 region was verified by PCR using the primers cgp1-E/F and cgp2-E/F, respectively.

The plasmid pK19*mobsacB*-*P_tuf_*-*dxs* was constructed to replace the native *dxs* promoter with the *tuf* promoter region from *C. glutamicum* WT as described earlier [42]. The promoter exchange was verified by PCR using the primers *dxs E* and 33, and sequencing of the PCR product.

### 4.5. Combinatorial Gene Assembly, Library Construction and Overexpression of Carotenogenic Genes

The combinatorial assembly of genes *crtW_Ba_* and *crtZ_Pa_* was performed with Gibson Assembly [44]. The *crtW* gene was amplified from the genomic DNA of *Brevundimonas aurantiaca* in a one-pot-PCR containing an equimolar mixture of forward primers (N1-N24) and a reverse primer (N49) (Table 5). The *crtZ* gene was amplified from the genomic DNA of *Pantoea ananatis* in a one-pot-PCR containing an equimolar mixture of forward primers (N25-N48) and a reverse primer (N50) (Table 5). PCR products of both genes were gel-extracted (Macherey-Nagel) and cloned in *Bam*HI-restricted pSH1 applying Gibson Assembly. The transformation of *E. coli* DH5α was done as described above. 1/10 of the transformed cells were plated on selective agar plates for colony number calculation while 9/10 of the transformants were grown in selective liquid medium for plasmid isolation. Isolated plasmids were used for transformation of *C. glutamicum* strain BETA1.

Plasmids harboring a carotenogenic gene (general abbreviation *crt*), pECXT99A_*crt*, pEKEX3_*crt* or pVWEx1_*crt* allowed an IPTG-inducible overexpression of *crt*. The vector pSH1 expressed *crt* constitutively under the *tuf*-promoter (*P_tuf_*). The plasmids were constructed on the basis of pECXT99A [79] pEKEx3 [80] or pVWEx1 [81], respectively. Amplification of *crt* was achieved by polymerase chain reaction (PCR) from genomic DNA of *C. glutamicum* ATCC 13032, *P. ananatis*, *B. aurantiaca*, *B. bacteroides*, *B. vesicularis*, *F. pelagi*, and *S. astaxanthinifaciens*, respectively, that was prepared as described [86] using the respective primers (Table 5). The amplified products were cloned into the *Bam*HI restricted pEC-XT99A, pEKEx3, pVWEx1 or pSH1 plasmid DNA by Gibson assembly.

### 4.6. Extraction and Quantification of Carotenoids

For extracting carotenoids from *C. glutamicum*, 1 mL of the culture was harvested by centrifugation for 7 min at 14,000 rpm. Carotenoid pigments were extracted with 800 µL methanol:acetone (7:3) containing 0.05% BHT at 60 °C for 15 min with careful vortexing every 5 min. Cell debris was spun down for 7 min at 14,000 rpm and the supernatant was used for high performance liquid chromatography (HPLC) analysis. For HPLC the Agilent 1200 series system (Agilent Technologies Sales & Services GmbH & Co. KG, Waldbronn, Germany) was used. The UV/visible (Vis) spectrum was recorded with a diode array detector (DAD). The quantification of carotenoids was performed by the integration of the extracted wavelength chromatogram at λ_max_ 470 nm for every maximum and by the analysis of the appropriate UV/Vis profiles. Standard calibration curves were generated with lycopene (Sigma-Aldrich), β-carotene (Sigma-Aldrich), canthaxanthin (Sigma-Aldrich), zeaxanthin (Sigma-Aldrich) and astaxanthin (Sigma-Aldrich) to quantify carotenoid titers. All standards were dissolved in chloroform according to their solubility and diluted in methanol:acetone (7:3) containing 0.05% BHT.

As column system, a precolumn (10 × 4 mm MultoHigh 100 RP18-5, CS Chromatographie Service GmbH, Langerwehe, Germany) and a main column (ProntoSIL 200–5 C30, 250 × 4 mm, CS Chromatographie Service GmbH) were used. The HPLC protocol ensured a gradient elution for 10 min and a mobile phase composition of (A) methanol and (B) methanol/methyl tert-butyl ether/ethyl acetate (5:4:1) starting from 10% to 100% of eluent B, followed by 20 min of isocratic elution with 100% B. After that, the eluent composition was set back to 10% B for 3 min. The injection volume was 100 μL and the flow rate was kept constant at 1.4 mL/min.

## Figures and Tables

**Table 1:** Lycopene production by plasmid-free recombinant *C. glutamicum* strains. Cells were grown in glucose CGXII minimal medium for 24 h. Means and standard deviations of three replicates are given.

Name	Strain	Lycopene (mg·(g·CDW)^−1^)
LYC3	*crtY_e_Y_f_Eb* deletion mutant of *C. glutamicum* MB001	0.04 ± 0.01
LYC3-*P_tuf_dxs*	LYC3::*P_tuf_dxs*	0.09 ± 0.01
LYC4	LYC3::*P_tuf_crtEBI*	0.32 ± 0.01
LYC5	LYC4::*P_tuf_dxs*	0.43 ± 0.02

**Table 2:** β-Carotene production in recombinant *C. glutamicum* strains. Cells were grown in glucose CGXII minimal medium for 24 h induced by 1 mM isopropyl β-d-1-thiogalactopyranoside (IPTG). Means and standard deviations of three replicates are given.

Name	Strain	β-Carotene (mg·(g·CDW)^−1^)
BETA1	LYC5 (pEXEx3_*crtY_Pa_*)	5.2 ± 1.0
BETA2	LYC5 (pSH1_*crtY_Pa_*)	5.9 ± 0.8
BETA3	LYC5::*P_tuf__ crtY_Pa_*	6.5 ± 1.3

**Table 3:** Astaxanthin, canthaxanthin, and β-carotene production by strains overexpressing various combinations of *crtW* and *crtZ* genes. Titers, productivities, and final ODs are given as means and standard deviations (*n* = 3) after 24 h of cultivation in CGXII + 100 mM glucose. *B.a.*: *Brevundimonas aurantiaca*; *B.b.*: *Brevundimonas bacteroides*; *F.p.*: *Fulvimarina pelagi*; *S.a.*: *Sphingomonas astaxanthinifaciens*.

Strain Growth		Carotenoid Titer (mg·g^−1^·CDW)			Volumetric Productivity (mg·L^−1^·h^−1^)		
**BETA4 Transformed with**	**Final OD_600 nm_**	**Astaxanthin**	**Canthaxanthin**	**β-Carotene**	**Astaxanthin**	**Canthaxanthin**	**β-Carotene**
-	28 ± 1	<0.1	<0.1	11.7 ± 2.0	<0.1	<0.1	3.4 ± 0.5
(pSH1_*crtW_Bb_*) (pEC-XT_*crtZ_Bb_*)	21 ± 1	<0.1	<0.1	4.9 ± 0.4	<0.1	<0.1	1.1 ± 0.1
(pSH1_*crtW_Sa_*) (pEC-XT_*crtZ_Sa_*)	22 ± 2	< 0.1	0.3 ± 0.1	3.3 ± 0.5	<0.1	<0.1	0.8 ± 0.1
(pSH1_*crtW_Sa_*) (pEC-XT_*crtZ_Fp_*)	24 ± 1	0.7 ± 0.3	0.2 ± 0.1	1.8 ± 0.1	0.2 ± 0.1	<0.1	0.5 ± 0.1
(pSH1_*crtW_Ba_*) (pEC-XT_*crtZ_Fp_*)	22 ± 1	1.7 ± 0.3	0.1 ± 0.1	2.0 ± 0.5	0.4 ± 0.1	<0.1	0.4 ± 0.2
(pSH1_*crtW_Fp_*) (pEC-XT_*crtZ_Fp_*) = ASTA1	23 ± 1	1.6 ± 0.3	0.1 ± 0.1	0.3 ± 0.1	0.4 ± 0.1	<0.1	0.1 ± 0.1

**Table 4:** Strains and plasmids used in this study.

Strain; Plasmid	Relevant Characteristics	Reference
***C. glutamicum* Strains**		
WT	Wild type, ATCC 13032	[74]
MB001	prophage cured, genome reduced ATCC 13032	[72]
LYC3	*crtY_e_Y_f_Eb* deletion mutant of *C. glutamicum* MB001	[42]
LYC4	LYC3 derivative with an artificial operon containing *crtE, crtB*, and *crtI* under control of the *P_tuf_* promoter integrated into the chromosome	this work
LYC5	LYC4 derivative with *dxs* under control of the *P_tuf_* promoter integrated into the chromosome	this work
BETA1	LYC5 derivative (pEKEx3_ *crtY_Pa_*)	this work
BETA2	LYC5 derivative (pSH1_ *crtY_Pa_*)	this work
BETA3	LYC5 derivative with *crtY_Pa_* under control of the *P_tuf_* promoter integrated into the chromosome	this work
BETA4	*cg0725* deletion mutant of *C. glutamicum* BETA3	this work
ASTA1	*C. glutamicum* BETA4 carrying pSH1_*crtW1_Fp_* and pEC-XT_*crtZ_Fp_*	this work
**Other Strains**		
*E. coli* DH5α	F- *thi*-1 *endA1 hsdr17*(r-, m-) *supE44* Δ*lacU169* (Φ80*lacZ*ΔM15) *recA1 gyrA96*	[75]
*Pantoea ananatis*	Wild type, ATCC 33244, DSM 17873, Z96081	[76]
*Brevundimonas aurantiaca*	Wild type, ATCC 15266, DSM 4731, NR028889	[77]
*Brevundimonas bacteroides*	Wild type, ATCC 15254, DSM 4726, AJ227782	[49]
*Brevundimonas vesicularis*	Wild type, ATCC 11426, DSM 7226, LN681560	[78]
*Fulvimarina pelagi*	Wild type, ATCC BAA-666, DSM 15513, AY178860	[50]
*Sphingomonas astaxanthinifaciens*	Wild type, NBRC 102146, DSM 22298, AB277583	[52]
**Plasmids**		
pEC-XT99A (pEC-XT)	Tet^R^, *P_trc_lacI^q^*, pGA1 *oriV_Cg_*, *C. glutamicum*/*E. coli* expression shuttle vector	[79]
pEC-XT_*crtZ_Bb_*	pEC-XT derivative for IPTG-inducible expression of *crtZ* from *B. bacteroides* containing an artificial ribosome binding site	this work
pEC-XT_*crtZ_Bv_*	pEC-XT derivative for IPTG-inducible expression of *crtZ* from *B. vesicularis* containing an artificial ribosome binding site	this work
pEC-XT_*crtZ_Fp_*	pEC-XT derivative for IPTG-inducible expression of *crtZ* from *F. pelagi* containing an artificial ribosome binding site	this work
pEC-XT_*crtZ_Sa_*	pEC-XT derivative for IPTG-inducible expression of *crtZ* from *S. astaxanthinifaciens* containing an artificial ribosome binding site	this work
pEKEx3	Spec^R^, *P_tac_lacI^q^*, pBL1 *oriV_Cg_*, *C. glutamicum*/*E. coli* expression shuttle vector	[80]
pEKEx3_*crtY_Pa_*	pEKEx3 derivative for IPTG-inducible expression of *crtY* from *P. ananatis* containing an artificial ribosome binding site	this work
pVWEx1	Km^R^, *P_tac_lacI^q^*, pHM519 *oriV_Cg_*, *C. glutamicum*/*E. coli* expression shuttle vector	[81]
pSH1	Km^R^, *P_tuf_*, pHM519 *oriV_Cg_*, *C. glutamicum*/*E. coli* expression shuttle vector	this work
pSH1_*crtY_Pa_*	pSH1 derivative for constitutive expression of *crtY* from *P. ananatis* containing an artificial ribosome binding site	this work
pSH1_*crtW_Ba__crtZ_Pa_*	pSH1 derivative for constitutive expression of *crtW* from *B. aurantiaca* and *crtZ* from *P. ananatis* containing artificial ribosome binding sites	this work
pSH1_*crtW_Ba_*	pSH1 derivative for constitutive expression of *crtW* from *B. aurantiaca* containing an artificial ribosome binding site	this work
pSH1_*crtW_Bb_*	pSH1 derivative for constitutive expression of *crtW* from *B. bacteroides* containing an artificial ribosome binding site	this work
pSH1_*crtW1_Bv_*	pSH1 derivative for constitutive expression of *crtW* from *B. vesicularis* containing an artificial ribosome binding site	this work
pSH1_*crtW2_Bv_*	pSH1 derivative for constitutive expression of *crtW* from *B. vesicularis* containing an artificial ribosome binding site	this work
pSH1_*crtW1_Fp_*	pSH1 derivative for constitutive expression of *crtW* from *F. pelagi* containing an artificial ribosome binding site	this work
pSH1_*crtW2_Fp_*	pSH1 derivative for constitutive expression of *crtW* from *F. pelagi* containing an artificial ribosome binding site	this work
pSH1_*crtW3_Fp_*	pSH1 derivative for constitutive expression of *crtW* from *F. pelagi* containing an artificial ribosome binding site	this work
pSH1_*crtW_Sa_*	pSH1 derivative for constitutive expression of *crtW* from *S. astaxanthinifaciens* containing an artificial ribosome binding site	this work
pK19*mobsacB*	Km^R^; *E. coli*/*C. glutamicum* shuttle vector for construction of insertion and deletion mutants in *C. glutamicum* (pK18 *oriV_Ec_ sacB lacZα*)	[82]
pK19*mobsacB-cg0725*	pK19*mobsacB* with a *cg0725* deletion construct	-
pK19*mobsacB*-*P_tuf_*-*dxs*	pK19*mobsacB* derivativ*e* with a *tuf* promoter region (200 bp upstream of the coding sequence of the *tuf* gene(cg0587) construct for the promoter exchange of *dxs*	[42]
pK19*mobsacB*-Int*crtEBI*	pK19*mobsacB* derivative containing the artificial operon *crtE_crtBI* under the control of the *P_tuf_* promoter with an addition ribosome binding site in front of *crtB* for integration in the cgp2 cured region of *C. glutamicum* MB001	this work
pVWEx1-*crtEBI*	pVWEx1 derivative for IPTG-inducible expression of *crtE*, *crtB* and *crtI* from *C. glutamicum* containing artificial ribosome binding sites in front of *crtE* and *crtBI*	[38]
pK19*mobsacB*-Int*crtY*	pK19*mobsacB* derivative containing *crtY* of *Pantoea ananatis* under the control of the *P_tuf_* promoter for integration in the cgp1 cured region of *C. glutamicum* MB001	this work

**Table 5:** Oligonucleotides used in this study.

Oligonucleotide	Sequence (5'→3')
N1	*CATGCCTGCAGGTCGACTCTAGAG***GAAAGGAGGCCCTTCAG**ATGACCGCCGCCGTCGCCGAG
N2	*CATGCCTGCAGGTCGACTCTAGAG***GAAAGGAGGCCCTTCAG**GTGACCGCCGCCGTCGCCGAG
N3	*CATGCCTGCAGGTCGACTCTAGAG***GAAAGGAGGCCCTTC**ATGACCGCCGCCGTCGCCGAG
N4	*CATGCCTGCAGGTCGACTCTAGAG***GAAAGGAGGCCCTT**CGTGACCGCCGCCGTCGCCGAG
N5	*CATGCCTGCAGGTCGACTCTAGAG***GAAAGGAGGCCC**ATGACCGCCGCCGTCGCCGAG
N6	*CATGCCTGCAGGTCGACTCTAGAG***GAAAGGAGGCCC**GTGACCGCCGCCGTCGCCGAG
N7	*CATGCCTGCAGGTCGACTCTAGAG***GAAAGAAGGCCCTTCAG**ATGACCGCCGCCGTCGCCGAG
N8	*CATGCCTGCAGGTCGACTCTAGAG***GAAAGAAGGCCCTTCAG**GTGACCGCCGCCGTCGCCGAG
N9	*CATGCCTGCAGGTCGACTCTAGAG***GAAAGAAGGCCCTTC**ATGACCGCCGCCGTCGCCGAG
N10	*CATGCCTGCAGGTCGACTCTAGAG***GAAAGAAGGCCCTTC**GTGACCGCCGCCGTCGCCGAG
N11	*CATGCCTGCAGGTCGACTCTAGAG***GAAAGAAGGCCC**ATGACCGCCGCCGTCGCCGAG
N12	*CATGCCTGCAGGTCGACTCTAGAG***GAAAGAAGGCCC**GTGACCGCCGCCGTCGCCGAG
N13	*CATGCCTGCAGGTCGACTCTAGAG***GAATGGAGGCCCTTCAG**ATGACCGCCGCCGTCGCCGAG
N14	*CATGCCTGCAGGTCGACTCTAGAG***GAATGGAGGCCCTTCAG**GTGACCGCCGCCGTCGCCGAG
N15	*CATGCCTGCAGGTCGACTCTAGAG***GAATGGAGGCCCTTC**ATGACCGCCGCCGTCGCCGAG
N16	*CATGCCTGCAGGTCGACTCTAGAG***GAATGGAGGCCCTTC**GTGACCGCCGCCGTCGCCGAG
N17	*CATGCCTGCAGGTCGACTCTAGAG***GAATGGAGGCCC**ATGACCGCCGCCGTCGCCGAG
N18	*CATGCCTGCAGGTCGACTCTAGAG***GAATGGAGGCCC**GTGACCGCCGCCGTCGCCGAG
N19	*CATGCCTGCAGGTCGACTCTAGAG***GAATGAAGGCCCTTCAG**ATGACCGCCGCCGTCGCCGAG
N20	*CATGCCTGCAGGTCGACTCTAGAG***GAATGAAGGCCCTTCAG**GTGACCGCCGCCGTCGCCGAG
N21	*CATGCCTGCAGGTCGACTCTAGAG***GAATGAAGGCCCTTC**ATGACCGCCGCCGTCGCCGAG
N22	*CATGCCTGCAGGTCGACTCTAGAG***GAATGAAGGCCCTTC**GTGACCGCCGCCGTCGCCGAG
N23	*CATGCCTGCAGGTCGACTCTAGAG***GAATGAAGGCCC**ATGACCGCCGCCGTCGCCGAG
N24	*CATGCCTGCAGGTCGACTCTAGAG***GAATGAAGGCCC**GTGACCGCCGCCGTCGCCGAG
N25	*AACTGCCACACGAAC***GAAAGGAGGCCCTTCAG**ATGTTGTGGATTTGGAATGCCCTGATC
N26	*AACTGCCACACGAAC***GAAAGGAGGCCCTTCAG**GTGTTGTGGATTTGGAATGCCCTGATC
N27	*AACTGCCACACGAAC***GAAAGGAGGCCCTTC**ATGTTGTGGATTTGGAATGCCCTGATC
N28	*AACTGCCACACGAAC***GAAAGGAGGCCCTTC**GTGTTGTGGATTTGGAATGCCCTGATC
N29	*AACTGCCACACGAAC***GAAAGGAGGCCC**ATGTTGTGGATTTGGAATGCCCTGATC
N30	*AACTGCCACACGAAC***GAAAGGAGGCCC**GTGTTGTGGATTTGGAATGCCCTGATC
N31	*AACTGCCACACGAAC***GAAAGAAGGCCCTTCAG**ATGTTGTGGATTTGGAATGCCCTGATC
N32	*AACTGCCACACGAAC***GAAAGAAGGCCCTTCAG**GTGTTGTGGATTTGGAATGCCCTGATC
N33	*AACTGCCACACGAAC***GAAAGAAGGCCCTTC**ATGTTGTGGATTTGGAATGCCCTGATC
N34	*AACTGCCACACGAAC***GAAAGAAGGCCCTTC**GTGTTGTGGATTTGGAATGCCCTGATC
N35	*AACTGCCACACGAAC***GAAAGAAGGCCC**ATGTTGTGGATTTGGAATGCCCTGATC
N36	*AACTGCCACACGAAC***GAAAGAAGGCCC**GTGTTGTGGATTTGGAATGCCCTGATC
N37	*AACTGCCACACGAAC***GAATGGAGGCCCTTCAG**ATGTTGTGGATTTGGAATGCCCTGATC
N38	*AACTGCCACACGAAC***GAATGGAGGCCCTTCAG**GTGTTGTGGATTTGGAATGCCCTGATC
N39	*AACTGCCACACGAAC***GAATGGAGGCCCTTC**ATGTTGTGGATTTGGAATGCCCTGATC
N40	*AACTGCCACACGAAC***GAATGGAGGCCCTTC**GTGTTGTGGATTTGGAATGCCCTGATC
N41	*AACTGCCACACGAAC***GAATGGAGGCCC**ATGTTGTGGATTTGGAATGCCCTGATC
N42	*AACTGCCACACGAAC***GAATGGAGGCCC**GTGTTGTGGATTTGGAATGCCCTGATC
N43	*AACTGCCACACGAAC***GAATGAAGGCCCTTCAG**ATGTTGTGGATTTGGAATGCCCTGATC
N44	*AACTGCCACACGAAC***GAATGAAGGCCCTTCAG**GTGTTGTGGATTTGGAATGCCCTGATC
N45	*AACTGCCACACGAAC***GAATGAAGGCCCTTC**ATGTTGTGGATTTGGAATGCCCTGATC
N46	*AACTGCCACACGAAC***GAATGAAGGCCCTTC**GTGTTGTGGATTTGGAATGCCCTGATC
N47	*AACTGCCACACGAAC***GAATGAAGGCCC**ATGTTGTGGATTTGGAATGCCCTGATC
N48	*AACTGCCACACGAAC***GAATGAAGGCCC**GTGTTGTGGATTTGGAATGCCCTGATC
N49	*GTTCGTGTGGCAGTT*TTAGTGGTGGTGGTGGTGGTGAGACTCGCCGCGCCACAGACG
N50	*ATTCGAGCTCGGTACCCGGGGATC*TTACTTTTCGAACTGTGGGTGGGACCACTTCCCGGATGCGGGCTC
BaW1	*CATGCCTGCAGGTCGACTCTAGAG***GAAAGGAGGCCCTTCAG**ATGACCGCCGCCGTCGCCGAG
BaW2	*CGGTACCCGGGGATC*TCAAGACTCGCCGCGCCAC
BbW1	*CATGCCTGCAGGTCGACTCTAGAG***GAAAGGAGGCCCTTCAG**ATGACGCGGGAACGCCA
BbW2	*ATTCGAGCTCGGTACCCGGGGATC*TTAGAGACGTTCGCTACGC
BbZ1	*ATGGAATTCGAGCTCGGTACCCGGG***GAAAGGAGGCCCTTCAG**ATGACGATCGTCTGGTTCAC
BbZ2	*GCATGCCTGCAGGTCGACTCTAGAGGATC*TTACTCGGCCGGGATGTCC
BvW1	*CATGCCTGCAGGTCGACTCTAGAG***GAAAGGAGGCCCTTCAG**ATGGGGCAAGCGAACAG
BvW2	*CATGCCTGCAGGTCGACTCTAGAG***GAAAGGAGGCCCTTCAG**ATGCGGCAAGCGAACAG
BvW3	*ATTCGAGCTCGGTACCCGGGGAT*CCTAGCTGAACAAACTCCACCAG
BvZ1	*ATGGAATTCGAGCTCGGTACCCGGG***GAAAGGAGGCCCTTCAG**ATGTCCTGGCCGACGATG
BvZ2	*GCATGCCTGCAGGTCGACTCTAGAGGATC*TTAGGCGCCGTTGCTGGAT
FpW1	*CATGCCTGCAGGTCGACTCTAGAG***GAAAGGAGGCCCTTCAG**ATGACCCTCAGCCCAACCTC
FpW2	*CATGCCTGCAGGTCGACTCTAGAG***GAAAGGAGGCCCTTCAG**ATGAGACCCTACCAAACGACG
FpW3	*CATGCCTGCAGGTCGACTCTAGAG***GAAAGGAGGCCCTTCAG**ATGCATGGTTCGCTGGC
FpW4	*ATTCGAGCTCGGTACCCGGGGATC*TTAGGACTGGCGAGTATGCG
FpZ1	*ATGGAATTCGAGCTCGGTACCCGGG***GAAAGGAGGCCCTTCAG**ATGACGATCTGGACTCTCTACTAC
FpZ2	*GCATGCCTGCAGGTCGACTCTAGAGGATC*TTACCGAACCGGCGCGT
SaW1	*CATGCCTGCAGGTCGACTCTAGAG***GAAAGGAGGCCCTTCAG**ATGGCCCCCATGCTCAGTG
SaW2	*ATTCGAGCTCGGTACCCGGGGATC*TTAGGCGGGAAGCGCAAG
SaZ1	*ATGGAATTCGAGCTCGGTACCCGGG***GAAAGGAGGCCCTTCAG**ATGTCCTGGCCTGCCG
SaZ2	*GCATGCCTGCAGGTCGACTCTAGAGGATC*TTAGGCCCGCTCCTCGTG
pSH1 fw	ACCGGCTCCAGATTTATCAG
pVWEx/ pSH1 rv	ATCTTCTCTCATCCGCCA
pEC-XT fw	AATACGCAAACCGCCTCTCC
pEC-XT rv	TACTGCCGCCAGGCAAATTC
pV_*P_tuf_* -fw	*CGGAATCTTGCACGCCCT*TGGCCGTTACCCTGCGAATG
pV_*P_tuf_* -rv	*CTGCAGGCATGCAAGCTT*TGTATGTCCTCCTGGACTTC
pV1-fw	*GAAGTCCAGGAGGACATACA*AAGCTTGCATGCCTGCAG
pV6962-rv	*CATTCGCAGGGTAACGGCCA*AGGGCGTGCAAGATTCCG
*cg0725*-A	*GCAGGTCGACTCTAGAGGATCCCC*GCGCGAAGATTTGATGGG
*cg0725*-B	*CCCATCCACCCCGGGTAAACA*TTCCTGCATATTCAGCATAGTAATC
*cg0725*-C	*TGTTTACCCGGGGTGGATGGG*TCCCTTAATAATGCACCATGGC
*cg0725*-D	*CCAGTGAATTCGAGCTCGGTACCCC*TTGTCACCACAGCACTACT
*cg0725*-E	GCGCGAAGATTTGATGGG
*cg0725*-F	ACTTGTCACCACAGCACTAC
*crtY*-Int1	GCAGGTCGACTCTAGAGGATCCCCCAGTGAAGGATCGGTGCG
*crtY*-Int2	CATTCGCAGGGTAACGGCCACCTATCTGCTGGCCGGTG
*crtY*-Int3	CACCGGCCAGCAGATAGGTGGCCGTTACCCTGCGAATG
*crtY*-Int4	CAGATCATAATGCGGTTGCATTGTATGTCCTCCTGGACTTC
*crtY*-Int5	GAAGTCCAGGAGGACATACAATGCAACCGCATTATGATCTG
*crtY*-Int6	TCTTACTACTTGCGCTAGGTACAGTTAACGATGAGTCGTCATAATGG
*crtY*-Int7	CCATTATGACGACTCATCGTTAACTGTACCTAGCGCAAGTAGTAAGA
*crtY*-Int8	CCAGTGAATTCGAGCTCGGTACCCCTGCTCATCCTTCAACAACGT
cgp1-E	GTGGTGCTCGAGAACATAAG
cgp1-F	CGGTCACCCGTAACAATCAG
*crtEBI*-Int1	*GCAGGTCGACTCTAGAGGATCCCC*GTGCTTCGCATCGTCTATGTC
*crtEBI*-Int2	CATTCGCAGGGTAACGGCCAATAGTTGGGGGAATTTATAAGGATTTG
*crtEBI*-Int3	CAAATCCTTATAAATTCCCCCAACTATTGGCCGTTACCCTGCGAATG
*crtEBI*-Int4	GATTGTCATGCCATTGTCCATTGTATGTCCTCCTGGACTTC
*crtEBI*-Int5	GAAGTCCAGGAGGACATACAATGGACAATGGCATGACAATC
*crtEBI*-Int6	CTAATGGACGGTGAAGTATCATTTATGTTAATGATCGTATGAGGTCTTTTGAG
*crtEBI*-Int7	CTCAAAAGACCTCATACGATCATTAACATAAATGATACTTCACCGTCCATTAG
*crtEBI*-Int8	*CCAGTGAATTCGAGCTCGGTACCC*CGCCGTATGTAACAAGATTTG
Cgp2-E	TCGCACCATCTACGACAACC
Cgp2-F	CTACGAAGCTGACGCCGAAG

Sequence in bold: artificial ribosome binding site; sequence underlined: tag site; sequence in italics: linker sequence for hybridization.

## Abbreviations

The following abbreviations are used in this manuscript:

TIR	Translation Initiation Rate
MDPI	Multidisciplinary Digital Publishing Institute
DOAJ	Directory of open access journals
TLA	Three letter acronym
LD	Linear dichroism

## References

[1] Cooper D.A., Eldridge A.L., Peters J.C. (1999). Dietary carotenoids and certain cancers, heart disease, and age-related macular degeneration: A review of recent research. Nutr. Rev..

[2] Krinsky N.I., Johnson E.J. (2005). Carotenoid actions and their relation to health and disease. Mol. Asp. Med..

[3] Ye V.M., Bhatia S.K. (2012). Pathway engineering strategies for production of beneficial carotenoids in microbial hosts. Biotechnol. Lett..

[4] BBC Research The Global Market for Carotenoids. http://www.bccresearch.com/market-research/food-and-beverage/carotenoids-global-market-fod025d.html.

[5] Belviranli M., Okudan N., Lamprecht M. (2015). Well-Known Antioxidants and Newcomers in Sport Nutrition: Coenzyme Q10, Quercetin, Resveratrol, Pterostilbene, Pycnogenol and Astaxanthin. Antioxidants in Sport Nutrition.

[6] Gassel S., Schewe H., Schmidt I., Schrader J., Sandmann G. (2013). Multiple improvement of astaxanthin biosynthesis in *Xanthophyllomyces dendrorhous* by a combination of conventional mutagenesis and metabolic pathway engineering. Biotechnol. Lett..

[7] Liu X., Osawa T. (2007). *Cis* astaxanthin and especially 9-*cis* astaxanthin exhibits a higher antioxidant activity in vitro compared to the all-*trans* isomer. Biochem. Biophys. Res. Commun..

[8] Hussein G., Nakamura M., Zhao Q., Iguchi T., Goto H., Sankawa U., Watanabe H. (2005). Antihypertensive and neuroprotective effects of astaxanthin in experimental animals. Biol. Pharm. Bull..

[9] Giuffrida D., Sutthiwong N., Dugo P., Donato P., Cacciola F., Girard-Valenciennes E., le Mao Y., Monnet C., Fouillaud M., Caro Y. (2016). Characterisation of the C50 carotenoids produced by strains of the cheese-ripening bacterium *Arthrobacter arilaitensis*. Int. Dairy J..

[10] Chen C.W., Hsu S.H., Lin M.T., Hsu Y.H. (2015). Mass production of C50 carotenoids by *Haloferax mediterranei* in using extruded rice bran and starch under optimal conductivity of brined medium. Bioprocess Biosyst. Eng..

[11] Furubayashi M., Ikezumi M., Takaichi S., Maoka T., Hemmi H., Ogawa T., Saito K., Tobias A.V., Umeno D. (2015). A highly selective biosynthetic pathway to non-natural C50 carotenoids assembled from moderately selective enzymes. Nat. Commun..

[12] Umeno D., Arnold F.H. (2003). A C35 carotenoid biosynthetic pathway. Appl. Environ. Microbiol..

[13] Takaichi S., Sandmann G., Schnurr G., Satomi Y., Suzuki A., Misawa N. (1996). The carotenoid 7,8-dihydro-y end group can be cyclized by the lycopene cyclases from the bacterium *Erwinia uredovora* and the higher plant *Capsicum annuum*. Eur. J. Biochem..

[14] Fassett R.G., Coombes J.S. (2012). Astaxanthin in cardiovascular health and disease. Molecules.

[15] Ohgami K., Shiratori K., Kotake S., Nishida T., Mizuki N., Yazawa K., Ohno S. (2003). Effects of astaxanthin on lipopolysaccharide-induced inflammation in vitro and in vivo. Investig. Ophthalmol. Vis. Sci..

[16] Baralic I., Andjelkovic M., Djordjevic B., Dikic N., Radivojevic N., Suzin-Zivkovic V., Radojevic-Skodric S., Pejic S. (2015). Effect of Astaxanthin Supplementation on Salivary IgA, Oxidative Stress, and Inflammation in Young Soccer Players. Evid. Based. Complement. Altern. Med..

[17] Li J., Zhu D., Niu J., Shen S., Wang G. (2011). An economic assessment of astaxanthin production by large scale cultivation of *Haematococcus pluvialis*. Biotechnol. Adv..

[18] Zhu F., Zhong X., Hu M., Lu L., Deng Z., Liu T. (2014). In vitro Reconstitution of Mevalonate Pathway and targeted engineering of farnesene overproduction in *Escherichia coli*. Biotechnol. Bioeng..

[19] Capelli B., Bagchi D., Cysewski G. (2013). Synthetic astaxanthin is significantly inferior to algal-based astaxanthin as an antioxidant and may not be suitable as a human nutraceutical supplement. Nutrafoods.

[20] Lee P.C., Schmidt-Dannert C. (2002). Metabolic engineering towards biotechnological production of carotenoids in microorganisms. Appl. Microbiol. Biotechnol..

[21] George B., Synnove L.J., Hanspeter P. (2004). Carotenoids Handbook.

[22] Cutzu R., Coi A., Rosso F., Bardi L., Ciani M., Budroni M., Zara G., Zara S., Mannazzu I. (2013). From crude glycerol to carotenoids by using a *Rhodotorula glutinis* mutant. World J. Microbiol. Biotechnol..

[23] Kinoshita S., Udaka S., Shimono M. (1957). Studies on the amino acid fermentation. Production of *L*-glutamic acid by various microorganisms. J. Gen. Appl. Microbiol..

[24] Zahoor A., Otten A., Wendisch V.F. (2014). Metabolic engineering of *Corynebacterium glutamicum* for glycolate production. J. Biotechnol..

[25] Schneider J., Wendisch V.F. (2010). Putrescine production by engineered *Corynebacterium glutamicum*. Appl. Microbiol. Biotechnol..

[26] Blombach B., Eikmanns B.J. (2011). Current knowledge on isobutanol production with *Escherichia coli*, *Bacillus subtilis* and *Corynebacterium glutamicum*. Bioeng. Bugs.

[27] Frohwitter J., Heider S.A., Peters-Wendisch P., Beekwilder J., Wendisch V.F. (2014). Production of the sesquiterpene (+)-valencene by metabolically engineered *Corynebacterium glutamicum*. J. Biotechnol..

[28] Heider S.A., Peters-Wendisch P., Wendisch V.F., Beekwilder J., Brautaset T. (2014). Metabolic engineering for the microbial production of carotenoids and related products with a focus on the rare C50 carotenoids. Appl. Microbiol. Biotechnol..

[29] Kinoshita S., Tanaka K., Yamada K., Kinoshita S., Tsunoda T., Aida K. (1972). Glutamic acid. The Microbial Production of Amino Acids.

[30] Blombach B., Seibold G.M. (2010). Carbohydrate metabolism in *Corynebacterium glutamicum* and applications for the metabolic engineering of *L*-lysine production strains. Appl. Microbiol. Biotechnol..

[31] Meiswinkel T.M., Rittmann D., Lindner S.N., Wendisch V.F. (2013). Crude glycerol-based production of amino acids and putrescine by *Corynebacterium glutamicum*. Bioresour. Technol..

[32] Gopinath V., Meiswinkel T.M., Wendisch V.F., Nampoothiri K.M. (2011). Amino acid production from rice straw and wheat bran hydrolysates by recombinant pentose-utilizing *Corynebacterium glutamicum*. Appl. Microbiol. Biotechnol..

[33] Uhde A., Youn J.W., Maeda T., Clermont L., Matano C., Kramer R., Wendisch V.F., Seibold G.M., Marin K. (2013). Glucosamine as carbon source for amino acid-producing *Corynebacterium glutamicum*. Appl. Microbiol. Biotechnol..

[34] Matano C., Uhde A., Youn J.W., Maeda T., Clermont L., Marin K., Kramer R., Wendisch V.F., Seibold G.M. (2014). Engineering of *Corynebacterium glutamicum* for growth and *L*-lysine and lycopene production from *N*-acetyl-glucosamine. Appl. Microbiol. Biotechnol..

[35] Tsuchidate T., Tateno T., Okai N., Tanaka T., Ogino C., Kondo A. (2011). Glutamate production from beta-glucan using Endoglucanase-secreting *Corynebacterium glutamicum*. Appl. Microbiol. Biotechnol..

[36] Seibold G., Auchter M., Berens S., Kalinowski J., Eikmanns B.J. (2006). Utilization of soluble starch by a recombinant *Corynebacterium glutamicum* strain: Growth and lysine production. J. Biotechnol..

[37] Heider S.A., Peters-Wendisch P., Wendisch V.F. (2012). Carotenoid biosynthesis and overproduction in *Corynebacterium glutamicum*. BMC Microbiol..

[38] Heider S.A., Peters-Wendisch P., Netzer R., Stafnes M., Brautaset T., Wendisch V.F. (2014). Production and glucosylation of C50 and C40 carotenoids by metabolically engineered *Corynebacterium glutamicum*. Appl. Microbiol. Biotechnol..

[39] Heider S.A., Wendisch V.F. (2015). Engineering microbial cell factories: Metabolic engineering of *Corynebacterium glutamicum* with a focus on non-natural products. Biotechnol. J..

[40] Krubasik P., Kobayashi M., Sandmann G. (2001). Expression and functional analysis of a gene cluster involved in the synthesis of decaprenoxanthin reveals the mechanisms for C50 carotenoid formation. Eur. J. Biochem..

[41] Heider S.A., Peters-Wendisch P., Beekwilder J., Wendisch V.F. (2014). IdsA is the major geranylgeranyl pyrophosphate synthase involved in carotenogenesis in *Corynebacterium glutamicum*. FEBS J..

[42] Heider S.A., Wolf N., Hofemeier A., Peters-Wendisch P., Wendisch V.F. (2014). Optimization of the IPP precursor supply for the production of lycopene, decaprenoxanthin and astaxanthin by *Corynebacterium glutamicum*. Front. Bioeng. Biotechnol..

[43] Farasat I., Kushwaha M., Collens J., Easterbrook M., Guido M., Salis H.M. (2014). Efficient search, mapping, and optimization of multi-protein genetic systems in diverse bacteria. Mol. Syst. Biol..

[44] Gibson D.G., Young L., Chuang R.Y., Venter J.C., Hutchison C.A., Smith H.O. (2009). Enzymatic assembly of DNA molecules up to several hundred kilobases. Nat. Methods.

[45] Takano H., Agari Y., Hagiwara K., Watanabe R., Yamazaki R., Beppu T., Shinkai A., Ueda K. (2014). LdrP, a cAMP receptor protein/FNR family transcriptional regulator, serves as a positive regulator for the light-inducible gene cluster in the megaplasmid of *Thermus thermophilus*. Microbiology.

[46] Takano H., Mise K., Hagiwara K., Hirata N., Watanabe S., Toriyabe M., Shiratori-Takano H., Ueda K. (2015). The role and function of LitR, an AdoB_12_-bound light-sensitive regulator of *Bacillus megaterium* QM B1551, in the regulation of carotenoid production. J. Bacteriol..

[47] Tao L., Rouviere P.E., Cheng Q. (2006). A carotenoid synthesis gene cluster from a non-marine *Brevundimonas* that synthesizes hydroxylated astaxanthin. Gene.

[48] Scaife M.A., Ma C.A., Ninlayarn T., Wright P.C., Armenta R.E. (2012). Comparative analysis of beta-carotene hydroxylase genes for astaxanthin biosynthesis. J. Nat. Prod..

[49] Poindexter J.S. (1964). Biological Properties and Classification of the *Caulobacter* Group. Bacteriol. Rev..

[50] Cho J.C., Giovannoni S.J. (2003). Fulvimarina pelagi gen. nov., sp. nov., a marine bacterium that forms a deep evolutionary lineage of descent in the order “*Rhizobiales*”. Int. J. Syst. Evol. Microbiol..

[51] Asker D., Amano S., Morita K., Tamura K., Sakuda S., Kikuchi N., Furihata K., Matsufuji H., Beppu T., Ueda K. (2009). Astaxanthin dirhamnoside, a new astaxanthin derivative produced by a radio-tolerant bacterium, *Sphingomonas astaxanthinifaciens*. J. Antibiot..

[52] Asker D., Beppu T., Ueda K. (2007). *Sphingomonas astaxanthinifaciens* sp. nov., a novel astaxanthin-producing bacterium of the family Sphingomonadaceae isolated from Misasa, Tottori, Japan. FEMS Microbiol. Lett..

[53] Kang I., Oh H.M., Lim S.I., Ferriera S., Giovannoni S.J., Cho J.C. (2010). Genome sequence of *Fulvimarina pelagi* HTCC2506^T^, a Mn(II)-oxidizing alphaproteobacterium possessing an aerobic anoxygenic photosynthetic gene cluster and Xanthorhodopsin. J. Bacteriol..

[54] Knoll A., Bartsch S., Husemann B., Engel P., Schroer K., Ribeiro B., Stockmann C., Seletzky J., Buchs J. (2007). High cell density cultivation of recombinant yeasts and bacteria under non-pressurized and pressurized conditions in stirred tank bioreactors. J. Biotechnol..

[55] Aflalo C., Meshulam Y., Zarka A., Boussiba S. (2007). On the relative efficiency of two- vs. one-stage production of astaxanthin by the green alga *Haematococcus pluvialis*. Biotechnol. Bioeng..

[56] Landry A.P., Ding H. (2014). Redox Control of Human Mitochondrial Outer Membrane Protein MitoNEET [2Fe-2S] Clusters by Biological Thiols and Hydrogen Peroxide. J. Biol. Chem..

[57] Xie W., Liu M., Lv X., Lu W., Gu J., Yu H. (2014). Construction of a controllable beta-carotene biosynthetic pathway by decentralized assembly Strategy in *Saccharomyces Cerevisiae*. Biotechnol. Bioeng..

[58] Lemuth K., Steuer K., Albermann C. (2011). Engineering of a plasmid-free *Escherichia coli* strain for improvedin vivo biosynthesis of astaxanthin. Microb. Cell Fact..

[59] Steiger S., Sandmann G. (2004). Cloning of two carotenoid ketolase genes from *Nostoc punctiforme* for the heterologous production of canthaxanthin and astaxanthin. Biotechnol. Lett..

[60] Fraser P.D., Miura Y., Misawa N. (1997). In vitro characterization of astaxanthin biosynthetic enzymes. J. Biol. Chem..

[61] Dong S., Huang Y., Zhang R., Wang S., Liu Y. (2014). Four different methods comparison for extraction of astaxanthin from green alga *Haematococcus pluvialis*. Sci. World J..

[62] Scaife M.A., Burja A.M., Wright P.C. (2009). Characterization of cyanobacterial beta-carotene ketolase and hydroxylase genes in *Escherichia coli*, and their application for astaxanthin biosynthesis. Biotechnol. Bioeng..

[63] Tejera N., Cejas J.R., Rodriguez C., Bjerkeng B., Jerez S., Bolanos A., Lorenzo A. (2007). Pigmentation, carotenoids, lipid peroxides and lipid composition of skin of red porgy (*Pagrus pagrus*) fed diets supplemented with different astaxanthin sources. Aquaculture.

[64] Rodriguez-Saiz M., de la Fuente J.L., Barredo J.L. (2010). *Xanthophyllomyces dendrorhous* for the industrial production of astaxanthin. Appl. Microbiol. Biotechnol..

[65] Schmidt I., Schewe H., Gassel S., Jin C., Buckingham J., Humbelin M., Sandmann G., Schrader J. (2011). Biotechnological production of astaxanthin with *Phaffia rhodozyma/Xanthophyllomyces dendrorhous*. Appl. Microbiol. Biotechnol..

[66] Ni H., Chen Q.H., He G.Q., Wu G.B., Yang Y.F. (2008). Optimization of acidic extraction of astaxanthin from *Phaffia rhodozyma*. J. Zhejiang Univ. Sci. B.

[67] Monnet C., Loux V., Gibrat J.F., Spinnler E., Barbe V., Vacherie B., Gavory F., Gourbeyre E., Siguier P., Chandler M. (2010). The *Arthrobacter arilaitensis* Re117 genome sequence reveals its genetic adaptation to the surface of cheese. PLoS ONE.

[68] Brown N.L., Stoyanov J.V., Kidd S.P., Hobman J.L. (2003). The MerR family of transcriptional regulators. FEMS Microbiol. Rev..

[69] Lorenz R.T., Cysewski G.R. (2000). Commercial potential for *Haematococcus* microalgae as a natural source of astaxanthin. Trends Biotechnol..

[70] Bumbak F., Cook S., Zachleder V., Hauser S., Kovar K. (2011). Best practices in heterotrophic high-cell-density microalgal processes: Achievements, potential and possible limitations. Appl. Microbiol. Biotechnol..

[71] Jacobson G., Jolly S., Sedmak J., Skatrud T., Wasileski J. (1999). Astaxanthin over-Producing Strains of Phaffia Rhodozyma, Methods for their Cultivation, and their Use in Animal Feeds. U.S. Patent.

[72] Baumgart M., Unthan S., Ruckert C., Sivalingam J., Grunberger A., Kalinowski J., Bott M., Noack S., Frunzke J. (2013). Construction of a prophage-free variant of *Corynebacterium glutamicum* ATCC 13032 for use as a platform strain for basic research and industrial biotechnology. Appl. Environ. Microbiol..

[73] Eggeling L., Reyes O., Eggeling L., Bott M. (2005). Experiments. Handbook of Corynebacterium Glutamicum.

[74] Abe S., Takayarna K., Kinoshita S. (1967). Taxonomical studies on glutamic acid producing bacteria. J. Gen. Appl. Microbiol..

[75] Hanahan D. (1983). Studies on transformation of *Escherichia coli* with plasmids. J. Mol. Biol..

[76] Misawa N., Nakagawa M., Kobayashi K., Yamano S., Izawa Y., Nakamura K., Harashima K. (1990). Elucidation of the Erwinia uredovora carotenoid biosynthetic pathway by functional analysis of gene products expressed in *Escherichia coli*. J. Bacteriol..

[77] Abraham W.R., Strömpl C., Meyer H., Lindholst S., Moore E.R., Christ R., Vancanneyt M., Tindall B.J., Bennasar A., Smit J. (1999). Phylogeny and polyphasic taxonomy of Caulobacter species. Proposal of Maricaulis gen. nov. with Maricaulis maris (Poindexter) comb. nov. as the type species, and emended description of the genera Brevundimonas and Caulobacter. Int. J. Syst. Bacteriol..

[78] Busing K.H., Doll W., Freytag K. (1953). Bacterial flora of the medicinal leech. Arch. Mikrobiol..

[79] Kirchner O., Tauch A. (2003). Tools for genetic engineering in the amino acid-producing bacterium *Corynebacterium glutamicum*. J. Biotechnol..

[80] Stansen C., Uy D., Delaunay S., Eggeling L., Goergen J.L., Wendisch V.F. (2005). Characterization of a *Corynebacterium glutamicum* lactate utilization operon induced during temperature-triggered glutamate production. Appl. Environ. Microbiol..

[81] Peters-Wendisch P.G., Schiel B., Wendisch V.F., Katsoulidis E., Mockel B., Sahm H., Eikmanns B.J. (2001). Pyruvate carboxylase is a major bottleneck for glutamate and lysine production by *Corynebacterium glutamicum*. J. Mol. Microbiol. Biotechnol..

[82] Schäfer A., Tauch A., Jäger W., Kalinowski J., Thierbach G., Puhler A. (1994). Small mobilizable multi-purpose cloning vectors derived from the *Escherichia coli* plasmids pK18 and pK19: Selection of defined deletions in the chromosome of *Corynebacterium glutamicum*. Gene.

[83] Sambrook J., Russell D. (2001). Molecular Cloning: A Laboratory Manual.

[84] Van der Rest M.E., Lange C., Molenaar D. (1999). A heat shock following electroporation induces highly efficient transformation of *Corynebacterium glutamicum* with xenogeneic plasmid DNA. Appl. Microbiol. Biotechnol..

[85] Eggeling L., Bott M. (2005). Handbook of Corynebacterium Glutamicum.

[86] Eikmanns B.J., Rittmann D., Sahm H. (1995). Cloning, sequence analysis, expression, and inactivation of the *Corynebacterium glutamicum* icd gene encoding isocitrate dehydrogenase and biochemical characterization of the enzyme. J. Bacteriol..

